# Psychometric Comparisons of Benevolent and Corrective Humor across 22 Countries: The Virtue Gap in Humor Goes International

**DOI:** 10.3389/fpsyg.2018.00092

**Published:** 2018-02-09

**Authors:** Sonja Heintz, Willibald Ruch, Tracey Platt, Dandan Pang, Hugo Carretero-Dios, Alberto Dionigi, Catalina Argüello Gutiérrez, Ingrid Brdar, Dorota Brzozowska, Hsueh-Chih Chen, Władysław Chłopicki, Matthew Collins, Róbert Ďurka, Najwa Y. El Yahfoufi, Angélica Quiroga-Garza, Robert B. Isler, Andrés Mendiburo-Seguel, TamilSelvan Ramis, Betül Saglam, Olga V. Shcherbakova, Kamlesh Singh, Ieva Stokenberga, Peter S. O. Wong, Jorge Torres-Marín

**Affiliations:** ^1^Department of Psychology, Personality and Assessment, University of Zurich, Zurich, Switzerland; ^2^Faculty of Education, Health and Wellbeing, Institute of Psychology, University of Wolverhampton, Wolverhampton, United Kingdom; ^3^Department of Methodology of Behavioral Sciences, Faculty of Psychology, Centro de Investigación Mente, Cerebro, y Comportamiento, University of Granada, Granada, Spain; ^4^Federazione Nazionale Clown Dottori (FNC), Cesena, Italy; ^5^Department of Psychology, Faculty of Humanities and Social Sciences, University of Rijeka, Rijeka, Croatia; ^6^Institute of English, Faculty of Philology, University of Opole, Opole, Poland; ^7^College of Education, National Taiwan Normal University, Taipei, Taiwan; ^8^Department of English Studies, Jagiellonian University, Kraków, Poland; ^9^School of Electronics, Electrical Engineering and Computer Science, Queen's University Belfast, Belfast, United Kingdom; ^10^Department of Psychology, Faculty of Arts and Letters, Catholic University in Ružomberok, Ružomberok, Slovakia; ^11^Department of Psychology, Faculty of Letters and Human Sciences, Lebanese University, Beirut, Lebanon; ^12^Departamento Académico de Psicología, Universidad de Monterrey, San Pedro Garza García, Mexico; ^13^School of Psychology, University of Waikato, Hamilton, New Zealand; ^14^Facultad de Educación, Universidad Andrés Bello, Santiago, Chile; ^15^Department of Psychology, HELP University, Kuala Lumpur, Malaysia; ^16^Psychology Department, Üsküdar University, Istanbul, Turkey; ^17^Faculty of Psychology, Saint Petersburg State University, Saint Petersburg, Russia; ^18^Department of Humanities and Social Sciences, Indian Institute of Technology Delhi, New Delhi, India; ^19^Department of Psychology, Faculty of Education, Psychology and Art, University of Latvia, Riga, Latvia; ^20^Centre for Fundamental and Liberal Education, Universiti Malaysia Terengganu, Kuala Nerus, Malaysia; ^21^Department of Experimental Psychology, Faculty of Psychology, Centro de Investigación Mente, Cerebro y Comportamiento, University of Granada, Granada, Spain

**Keywords:** humor, virtue, cross-cultural comparisons, measurement invariance, positive psychology

## Abstract

Recently, two forms of virtue-related humor, benevolent and corrective, have been introduced. Benevolent humor treats human weaknesses and wrongdoings benevolently, while corrective humor aims at correcting and bettering them. Twelve marker items for benevolent and corrective humor (the BenCor) were developed, and it was demonstrated that they fill the gap between humor as temperament and virtue. The present study investigates responses to the BenCor from 25 samples in 22 countries (overall *N* = 7,226). The psychometric properties of the BenCor were found to be sufficient in most of the samples, including internal consistency, unidimensionality, and factorial validity. Importantly, benevolent and corrective humor were clearly established as two positively related, yet distinct dimensions of virtue-related humor. Metric measurement invariance was supported across the 25 samples, and scalar invariance was supported across six age groups (from 18 to 50+ years) and across gender. Comparisons of samples within and between four countries (Malaysia, Switzerland, Turkey, and the UK) showed that the item profiles were more similar within than between countries, though some evidence for regional differences was also found. This study thus supported, for the first time, the suitability of the 12 marker items of benevolent and corrective humor in different countries, enabling a cumulative cross-cultural research and eventually applications of humor aiming at the good.

## Introduction

Humor has been extensively studied in many areas of psychology, ranging from basic to applied research (for an overview, see Martin, 2007). In the area of individual differences in humor, different concepts of humor styles have been proposed, either as individual differences in humor behaviors (Craik et al., 1996) or in the functions of humor (Martin et al., 2003). A more recent approach emphasizes eight different comic styles that were derived from an interdisciplinary approach (Ruch et al., 2018a), namely fun, (benevolent) humor, nonsense, wit, irony, satire/corrective humor, sarcasm, and cynicism. The present investigation focuses on two comic styles, benevolent and corrective humor, which are historically, conceptually, and empirically related to virtue. The aim is to compare the 12 marker items of benevolent and corrective humor (created by Ruch, 2012) across different countries to investigate their psychometric properties across countries, age groups, and gender.

According to Ruch and Heintz (2016), benevolent and corrective humor are both morally valued and aim at doing good. Benevolent humor includes an accepting attitude toward the world and toward human weaknesses, and it treats them benevolently. It also includes being aware of one's surroundings and of everyday occurrences, which can then be reframed and commented on in a benevolent and humorous way. Corrective humor criticizes wrongdoings of both individuals and institutions, and it mocks them in order to improve them. Thus, it adds a moral goal to the criticism, which distinguishes corrective humor from pure mockery or aggressive forms of humor that lack this component. The connection of benevolent and corrective humor with morality and values can be traced back to their humanistic and philosophical roots, originating in England in the nineteenth century (for details, see Ruch and Heintz, 2016).

There are elements that benevolent and corrective humor share as well as elements where they differ. Both styles involve spotting incongruities in everyday life that are not inherently humorous, rather than processing and appreciating canned humor. Furthermore, these incongruities are processed playfully (not seriously) and they are treated humorously. Thus, in both styles the protagonist is attentive to what happens in his/her surroundings and realizes that deviations from expectations occur. This contributes to a large positive correlation between the two styles. However, in benevolent humor, the wrongdoing is not considered to be very important; for example, Nicolson (1946) suggested that humor observes human frailty indulgently, without bothering to correct it. In corrective humor, however, the difference between the real and the ideal is noticed, and funny comments are made to mock and to press someone to do the right thing. The two styles are opposite in this respect, thus reducing their overall positive correlation.

In line with these conceptualizations, the initial study (Ruch and Heintz, 2016) supported positive relationships of benevolent and corrective humor with several character strengths based on the VIA (Values in Action) classification of strengths and virtues (Peterson and Seligman, 2004). Specifically, benevolent humor uniquely related to character strengths assigned to the virtues of temperance (e.g., forgiveness), wisdom and knowledge (e.g., love of learning), transcendence (e.g., hope, humor), humanity (e.g., social intelligence), and justice (e.g., fairness). Of note, these relationships were robust when controlling for the sense of humor (as conceptualized by McGhee, 2010). By contrast, corrective humor was mostly uncorrelated with the strengths, except for positive correlations with creativity, bravery, and humor. Once mockery was controlled for, however, positive relationships emerged also with fairness and love of learning. This supports the notion that benevolent and corrective humor fill a virtue gap in humor by showing unique relationships to character strengths that serve to fulfill different virtues (such as humanity, justice, and wisdom/knowledge).

Investigating benevolent and corrective humor across several countries and languages is relevant for several reasons. First, despite the historical relevance of these two virtue-related humor styles, they have been neglected in psychological research. Establishing that the two styles can be found and distinguished across several countries would further support the relevance of the virtue gap in humor. Second, supporting the psychometric properties of the 12 marker items (or a subset thereof) would pave the way for international investigations on the nomological network of benevolent and corrective humor, as well as their predictors and virtue-relevant outcomes. Third, large-scale cross-cultural studies in the area of humor and virtues have been scarce (for exceptions, see Park et al., 2006; Proyer et al., 2009; McGrath, 2015, 2016), thus making the present study a valuable contribution to cross-cultural humor research and positive psychology more generally. Additionally, the large sample also allows comparing differences in benevolent and corrective humor across age groups and gender as two central demographic characteristics.

The present study investigates the psychometric properties of a set of 12 marker items for benevolent and corrective humor (the *BenCor*) within 25 samples from 22 countries. This includes descriptive statistics, reliability, measurement invariance, factorial validity, construct validity, profile similarity across the 12 marker items, as well as age and gender differences. Measurement invariance includes testing metric invariance (i.e., equal item loadings on the latent factor) and scalar invariance (i.e., equal item intercepts on the latent factor). Metric invariance is needed to compare the factors and slopes across the samples, and scalar invariance is needed to compare mean scores across the samples (see Chen, 2008). This allows evaluating the suitability of the BenCor across samples from different countries, across different age groups, and across gender.

## Materials and methods

### Samples

Inclusion criteria for participants were (a) an age of at least 18 years, (b) a reasonable command of the language in which the survey was conducted, and (c) the completion of all BenCor marker items. Participants who selected the same answer option for each item (e.g., answered “strongly agree” to all items) were excluded. Table 1 gives an overview of the resulting 25 BenCor samples in the 22 countries.

**Table 1:** Overview of the 25 BenCor Samples in the 22 Countries.

**Countries**	**Language**	***N***	***% Males***	**Age (*M*)**	**Age (*Mdn*.)**	**Primary sample type**	**Data collection**
Austria	German	350	32.6	39.15	40.00	Community	Online
Chile	Spanish	227	48.0	29.29	29.00	Community & students	Online
China (Guangzhou, Beijing)	Chinese	243	38.7	20.10	20.00	Community & students	Online
Costa Rica	Spanish	173	47.4	33.37	30.00	Community	Offline
Croatia	Croatian	350	54.9	21.27	21.00	Students	Offline
Germany	German	282	36.2	34.28	29.50	Community & students	Online
India	Hindi	198	49.5	26.36	23.00	Community	Offline
Italy	Italian	193	44.0	36.77	35.00	Community	Online
Latvia	Latvian	413	33.4	33.23	30.00	Community & students	Online
Lebanon	Arabic	260	37.7	25.26	21.00	Students	Offline
Malaysia	Malay	239	42.3	24.95	24.00	Students	Online
Malaysia (Terengganu)	Malay	199	50.3	24.45	21.00	Students	Offline
Mexico	Spanish	198	49.0	20.88	21.00	Students	Offline
New Zealand	English	221	41.6	34.21	31.00	Community	Online
Poland	Polish	458	30.0	33.97	32.00	Community & students	Online
Russia	Russian	201	49.8	30.24	25.00	Community & students	Online & offline
Slovakia	Slovak	400	29.0	25.79	22.00	Students	Online & offline
Spain	Spanish	209	46.4	22.55	21.00	Students	Offline
Switzerland (students)	German	313	32.6	24.95	24.00	Students	Online
Switzerland (general)	German	533	37.9	39.09	34.00	Community	Online
Taiwan	Chinese (trad.)	440	48.4	21.00	20.00	Students	Offline
Turkey (non-graduates)	Turkish	336	33.3	25.55	22.00	Community & students	Online
Turkey (university graduates)	Turkish	320	34.4	36.73	36.00	Community & students	Online
UK (mostly England)	English	269	35.3	31.19	25.00	Students	Online & offline
UK (Northern Ireland)	English	201	59.7	23.70	20.00	Students	Online

As shown in Table 1, sample sizes ranged from 173 (Costa Rica) to 533 (Switzerland, general community sample), with 7,226 participants overall. Gender was mostly balanced across samples (*M* = 40.2% males), with the percentages ranging from 29.0% males (Slovakia) to 59.7% males (Northern Ireland). The average age of the samples ranged from 20.10 years (China) to 39.15 years (Austria), with an overall mean of 28.73 years. The median age was lowest for China, Taiwan, and Northern Ireland (*Mdn* = 20.00 years), while it was highest for Austria (*Mdn* = 40.00 years). Thus, most of the samples comprised young to middle-aged adults. This is also reflected in the sample type, which were primarily students in 11 samples, primarily adults from the community in 6 samples, and both students and adults from the community in 8 samples. Finally, data collection was conducted online in 14 samples, offline in 8 samples, and both online and offline in 3 samples.

### Measures

The BenCor (Ruch, 2012) assesses benevolent and corrective humor with 6 marker items each (see Table 2). The marker items were derived from descriptions of humor and satire (corresponding to benevolent and corrective humor, respectively) based on literary and linguistic analyses (Schmidt-Hidding, 1963). These literary concepts were transformed into psychological traits, capturing individual differences in the propensity to engage in benevolent and corrective humor (for details, see Ruch et al., 2018a). A first psychometric analysis of the 12 marker items in a German-speaking sample (Ruch and Heintz, 2016) supported (a) the two-factor structure (based on a principal component analysis), (b) the assignment of each item to the corresponding factor, (c) internal consistencies (Cronbach's alpha 0.82 for benevolent and 0.84 for corrective humor), and (d) the criterion validity of the two sets of marker items in terms of character strengths. Recent studies further supported the construct validity (self-other agreement) and the criterion validity (in terms of personality, character strengths, and well-being) of the 12 marker items (Ruch et al., 2018a,b). The BenCor employs a seven-point Likert scale ranging from 1 (*strongly disagree*) to 7 (*strongly agree*).

**Table 2:** Overview of the 12 BenCor Items Marking Benevolent (Ben) and Corrective (Cor) Humor.

**No**.	**Humor**	**Items**
1	Ben	I am a realistic observer of human weaknesses, and my good-natured humor treats them benevolently.
3	Ben	When my humor is aimed at human weaknesses, I include both myself and others.
5	Ben	On a large and small scale, the world is not perfect, but with a humorous outlook on the world I can amuse myself at the adversities of life.
7	Ben	I accept the imperfection of human beings and my everyday life often gives me the opportunity to smile benevolently about it.
9	Ben	Humor is suitable for arousing understanding and sympathy for imperfections and the human condition.
11	Ben	Even when facing unpleasant events I can keep my distance and discover something amusing or funny in it.
2	Cor	I have a critical attitude toward arrogant and unfair people and my mockery serves to establish equality and justice.
4	Cor	I parody people's bad habits to fight the bad and foolish behavior.
6	Cor	When fellow humans or institutions demonstrate their superiority unjustified, I use biting humor to belittle them.
8	Cor	I caricature my fellow humans' wrongdoings in a funny way to gently urge them to change.
10	Cor	I like to ridicule moral badness to induce or increase a critical attitude in other people.
12	Cor	If the circumstances are not as they actually should be, I poke fun at these moral transgressions or societal wrongdoings, hoping to improve them in the long term.

Additionally, demographic information was collected from the participants, such as gender and age, and also further information such as nationality, language skills, and education. In some samples, additional measures were employed that are not relevant to the present study.

### Procedure

Each non-native English speaking co-author received a standardized package for the translation of the BenCor and the data collection. This included the English version of the 12 marker items (in some cases additional language versions were provided upon request), questionnaire instructions, descriptions of benevolent and corrective humor, the scoring key, the paper by Ruch and Heintz (2016), a description of the standardized translation/back-translation procedure (i.e., a translation to the local language and an independent back-translation into English), and a paper on guidelines for test translations (Van de Vijver and Hambleton, 1996). All item-translating co-authors had the opportunity to discuss their translations and the item contents with the first and second author to ensure that the items preserved their meaning in the translation. If a translation to the local language already existed, the co-authors were asked to check the applicability of the translation and to suggest adaptations if necessary. For example, the Spanish version (translated in Spain) was slightly adapted to fit to the Chilean and Costa Rican form of Spanish.

The online samples were collected by sending a link to the survey, which were hosted on different platforms (such as SurveyMonkey, Unipark, or Qualtrix). The offline samples were collected by asking participants (e.g., in libraries or classrooms) to complete the questionnaire in a paper-pencil version. These data were then manually entered into standardized data sheet (Excel or SPSS). Participants were recruited via different means, such as mailing lists, personal contacts, social media, the university campus, and thus comprise convenience samples. To analyze the data, they were either directly downloaded from online platforms or they were sent in the standardized data sheet to the first author. The 25 samples were collected in accordance with the local ethical guidelines, and participants provided either online or written informed consent in accordance with the Declaration of Helsinki.

After the data collection and initial data analyses, all co-authors completed a collaborator's form to provide details on the translated instrument, the sample description, the data collection procedure, and the interpretation of the data. For example, they reported which type of sample was investigated, the language skills and nationalities of the sample, how participants were approached, which mode of data collection was employed (i.e., online or offline), and whether any unexpected events occurred while collecting the data.

### Analyses

#### Reliability and validity

The internal consistencies of the samples are indicated by Cronbach's alpha. The factorial validity of the BenCor was tested in principal components analyses (PCA) with oblimin rotation and in confirmatory factor analyses (CFA). Based on the pattern matrix (factor loadings) of the PCA, Tucker's phi as an index of factor congruence was computed across the 12 items, separately for the benevolent and the corrective humor factor. According to Lorenzo-Seva and Ten Berge (2006), Tucker's phi coefficients ≥0.95 indicate equality and coefficients from 0.85 to 0.94 indicate a fair similarity of the factors. The CFA was computed with the *lavaan* package (Rosseel, 2012) in *R* (R Development Core, 2015). The robust MLM estimator (with Satorra-Bentler corrections) was employed for all CFA analyses. The following fit indices were evaluated using the recommended cut-offs by Schermelleh-Engel et al. (2003): χ^2^/*df* (good: ≤2, acceptable: ≤3), comparative fit index (CFI; good: ≥0.97, acceptable: ≥0.95), root mean square error of approximation (RMSEA; good: ≤0.05, acceptable: ≤0.08), and standardized root mean square residual (SRMR; good: ≤0.05, acceptable: ≤0.10). The one- and two-factor structure of the 12 BenCor marker items and the unidimensionality of benevolent and corrective humor (six marker items each) were investigated in CFAs. These analyses were conducted separately for each sample and across all samples.

Construct validity (discriminant validity) was assessed utilizing the average variance explained (AVE) calculation. According to Fornell and Larcker (1981), the AVE is computed by averaging the squared standardized loadings of each item on the factor. Discriminant validity can be supported if the square root of the AVE of each factor is larger than the correlation between the factors (the Fornell-Larcker criterion). To avoid biases due to measurement error, the Fornell-Larcker criterion was evaluated in the CFAs only (separate for each sample and across the 25 samples).

#### Measurement invariance

Measurement invariance was tested separately for benevolent and corrective humor using a multi-group CFA with the semTools package (semTools Contributors, 2015) in *R*. Metric invariance was tested by forcing all item loadings to be equal across groups. This model was then compared with the baseline model that allows a free estimation of the item loadings, comparing the difference in the CFI and the RMSEA. Changes of ≤|0.01| in the CFI and changes of ≤|0.015| in the RMSEA were used as cut-offs to indicate measurement invariance (based on the recommendations by Cheung and Rensvold, 1999; Chen, 2007). Similarly, scalar invariance was tested by forcing both the intercepts and the loadings to be equal across groups. In addition, partial measurement invariance at the item-level was investigated. A baseline model with free item loadings served as a comparison for models in which the item loadings (for metric invariance) and item intercepts (for scalar invariance) were constrained across the groups. This model was shown to be superior to a constrained-baseline model, in which each item is freed to test its differential functioning (see Stark et al., 2006). The CFI difference of ≤|0.01| was used to evaluate the partial measurement invariance of single items. Metric measurement invariance was tested across the 25 samples, across gender (*n* = 2,906 males and *n* = 4,312 females), and across six age groups: 18–20 years (*n* = 1,624), 21–24 years (*n* = 1,981), 25–29 years (*n* = 1,081), 30–39 years (*n* = 1,225), 40–49 years (*n* = 704), and 50+ years (*n* = 580). Additionally, scalar invariance was tested for gender and age.

#### Cross-sample comparisons

Similarities in the 12 marker items between the 25 samples were analyzed in terms of (a) means, (b) corrected item-total correlations (CITC), (c) multidimensional scaling of item-profile similarities, and (d) profile correlations across the 12 items. For the multidimensional scaling, the item means were analyzed using the alternating least squares scaling (ALSCAL) algorithm and Euclidian distances. These analyses were conducted for all samples, with additional analyses focusing on the samples that shared a language (i.e., English, German, and Spanish) as well as samples from the same country (i.e., Malaysia, Switzerland, Turkey, and the UK).

## Results

### Descriptive statistics of benevolent and corrective humor

Table 3 shows the descriptive statistics of the BenCor in the 25 samples.

**Table 3:** Psychometric characteristics and correlations with gender of the 25 BenCor samples in the 22 countries.

**Countries**	**Benevolent humor**					**Corrective humor**					***r***_**BenCor**_	
	***M***	***SD***	**α**	**φ**	***r*_gender_**	***M***	***SD***	**α**	**φ**	***r*_gender_**	**Scales**	**Factors**
Austria	5.28	0.87	0.76	0.99	−0.07	4.20	1.14	0.83	0.99	−0.20^***^	0.40^***^	0.34
Chile	5.24	1.12	0.76	0.99	−0.09	4.56	1.36	0.82	0.98	−0.21^**^	0.37^***^	0.30
China (Guangzhou, Beijing)	5.11	0.82	0.74	0.93	0.11	4.19	0.94	0.73	0.95	−0.25^***^	0.33^***^	0.24
Costa Rica	5.01	1.17	0.76	0.95	0.01	4.48	1.46	0.85	0.95	−0.20^**^	0.61^***^	0.47
Croatia	5.26	0.82	0.65	0.95	0.05	4.50	0.93	0.69	0.96	−0.08	0.32^***^	0.24
Germany	5.04	0.87	0.74	0.98	−0.10	4.10	1.23	0.85	0.97	−0.21^***^	0.49^***^	0.39
India	5.33	0.76	0.51	0.77	0.10	4.71	1.06	0.70	0.86	−0.02	0.50^***^	0.25
Italy	5.38	0.79	0.66	0.94	0.04	4.50	1.13	0.80	0.95	−0.19^**^	0.34^***^	0.25
Latvia	5.36	0.89	0.77	0.92	−0.04	4.26	1.12	0.78	0.92	−0.27^***^	0.49^***^	0.34
Lebanon	4.66	0.80	0.56	0.95	0.02	3.51	1.05	0.66	0.94	−0.11	0.32^***^	0.26
Malaysia	5.12	0.85	0.63	0.93	−0.12	3.99	1.13	0.73	0.90	−0.32^***^	0.45^***^	0.32
Malaysia (Terengganu)	5.29	0.80	0.58	0.85	−0.08	4.31	1.05	0.69	0.86	−0.11	0.54^***^	0.27
Mexico	5.25	0.86	0.62	0.97	0.05	3.87	1.12	0.71	0.96	−0.21^**^	0.35^***^	0.29
New Zealand	5.40	0.75	0.62	0.99	0.04	4.26	1.08	0.79	0.99	−0.12	0.28^***^	0.24
Poland	5.22	0.87	0.72	0.95	0.00	4.27	1.14	0.76	0.98	−0.22^***^	0.34^***^	0.24
Russia	5.04	0.86	0.60	0.93	0.00	3.60	1.07	0.72	0.91	−0.21^**^	0.21^**^	0.15
Slovakia	5.05	0.84	0.67	0.97	−0.06	4.10	1.10	0.77	0.98	−0.21^***^	0.48^***^	0.37
Spain	5.44	0.81	0.65	0.97	−0.05	4.21	1.21	0.79	0.99	−0.19^**^	0.28^***^	0.23
Switzerland (students)	5.14	0.81	0.80	–	−0.12^*^	4.23	1.06	0.83	–	−0.28^***^	0.45^***^	0.43
Switzerland (general)	4.98	0.83	0.74	1.00	−0.10^*^	4.09	1.09	0.81	0.99	−0.27^***^	0.53^***^	0.45
Taiwan	5.07	0.85	0.72	0.97	−0.14^**^	4.12	1.09	0.80	0.95	−0.38^***^	0.37^***^	0.30
Turkey (non-graduates)	4.87	1.03	0.67	0.89	−0.04	3.89	1.23	0.72	0.88	−0.20^***^	0.54^***^	0.34
Turkey (graduates)	4.90	0.85	0.50	0.80	0.02	3.96	1.15	0.68	0.86	−0.20^***^	0.45^***^	0.22
UK (mostly England)	5.11	0.87	0.69	0.99	−0.11	4.19	1.11	0.78	0.94	−0.22^***^	0.41^***^	0.28
UK (Northern Ireland)	5.33	0.76	0.60	0.94	−0.05	4.41	1.07	0.75	0.92	−0.18^*^	0.37^***^	0.24

*α, Cronbach's alpha (internal consistency); φ, Tucker's phi (factor congruence to the Swiss student sample based on the pattern matrix in the principal component analysis with oblimin rotation); gender coded as 1 = male, 2 = female*.

* *p < 0.05*.

** *p < 0.01*.

*** *p < 0.001*.

As shown in Table 3, the means for benevolent humor ranged from 4.66 (Lebanon) to 5.44 (Spain), with a mean across samples of 5.16 (*slightly agree*). The means for corrective humor ranged from 3.51 (Lebanon) to 4.71 (India), with a mean of 4.18 (*neither agree nor disagree*). Additionally, every sample had numerically higher scores in benevolent than in corrective humor. The means of benevolent and corrective humor correlated positively with one another across the samples [*r*_(25)_ = 0.67, *p* < 0.001].

Regarding the variance in benevolent humor, the standard deviations ranged from 0.75 (New Zealand) to 1.17 (Costa Rica), with a mean of 0.86. For corrective humor, the variance was numerically larger and ranged from 0.93 (Croatia) to 1.46 (Costa Rica), with a mean of 1.12. Thus, both benevolent and corrective humor created sufficient variance within each sample, with a tendency for corrective humor to elicit more varied responses. Similar to the mean scores, the standard deviations of benevolent and corrective humor were strongly positively correlated [*r*_(25)_ = 0.82, *p* < 0.001].

### Reliability

Next, the reliability of benevolent and corrective humor was investigated in each sample. As shown in Table 3, internal consistencies (Cronbach's alpha) of benevolent humor exceeded 0.60 in 21 of the 25 samples. Exceptions were India, Lebanon, Malaysia (Terengganu sample) and Turkey (graduate sample), in which internal consistencies ranged from 0.50 to 0.58. Across all samples, the median was 0.67. For corrective humor, all internal consistencies exceeded 0.60 (*Mdn* = 0.77). Thus, the internal consistencies were sufficient for corrective humor in all samples, and for benevolent humor in most samples.

Next, unidimensionality (or homogeneity) was tested in CFAs, separate for the six marker items of benevolent and corrective humor. Table 4 shows the resulting fit indices for each of the two CFA models in the 25 samples.

**Table 4:** Overview of the fit indices of confirmatory factor analyses of the 6 marker items (one-factor models indicating unidimensionality/homogeneity) separate for benevolent and corrective humor across the 25 BenCor samples in the 22 countries.

**Countries**	**Benevolent humor (*df* = 9)**					**Corrective humor (*df* = 9)**				
	**χ^2^**	**χ^2^/*df***	**CFI**	**RMSEA**	**SRMR**	**χ^2^**	**χ^2^/*df***	**CFI**	**RMSEA**	**SRMR**
Austria	16.22	1.80	0.97	0.05	0.03	28.31^**^	3.15	0.96	0.08	0.04
Chile	24.65^**^	2.74	0.93	0.09	0.05	24.53^**^	2.73	0.96	0.09	0.04
China	17.91^*^	1.99	0.95	0.06	0.05	22.12^**^	2.46	0.93	0.08	0.05
Costa Rica	3.83	0.43	1.00	0.00	0.02	7.48	0.83	1.00	0.00	0.02
Croatia	16.83	1.87	0.95	0.05	0.04	13.23	1.47	0.98	0.04	0.03
Germany	21.04^*^	2.34	0.95	0.07	0.04	18.16^*^	2.02	0.98	0.06	0.03
India	12.48	1.39	0.93	0.04	0.05	20.98^*^	2.33	0.92	0.08	0.05
Italian	18.51^*^	2.06	0.91	0.07	0.05	12.91	1.43	0.99	0.05	0.04
Latvia	16.91	1.88	0.98	0.05	0.03	52.26^***^	5.81	0.92	0.11	0.05
Lebanon	25.70^**^	2.86	0.84	0.08	0.05	33.43^***^	3.71	0.87	0.10	0.06
Malaysia	21.16^*^	2.35	0.86	0.08	0.05	11.01	1.22	0.99	0.03	0.03
Malaysia (Terengganu)	10.61	1.18	0.97	0.03	0.04	12.47	1.39	0.97	0.04	0.04
Mexico	11.92	1.32	0.96	0.04	0.05	8.44	0.94	1.00	0.00	0.03
New Zealand	18.49^*^	2.05	0.86	0.07	0.06	15.01	1.67	0.98	0.06	0.04
Poland	16.57	1.84	0.98	0.04	0.03	26.47^**^	2.94	0.97	0.07	0.03
Russia	20.66^*^	2.30	0.87	0.08	0.05	17.83^*^	1.98	0.96	0.07	0.05
Slovakia	22.88^**^	2.54	0.93	0.06	0.04	26.71^**^	2.97	0.96	0.07	0.04
Spain	16.85	1.87	0.93	0.07	0.05	11.84	1.32	0.99	0.04	0.03
Switzerland (community)	12.50	1.39	0.99	0.03	0.02	16.16	1.80	0.99	0.04	0.02
Switzerland (students)	6.59	0.73	1.00	0.00	0.02	6.07	0.67	1.00	0.00	0.02
Taiwan	31.62^***^	3.51	0.93	0.08	0.05	29.35^***^	3.26	0.97	0.07	0.04
Turkey (non-graduates)	21.33^*^	2.37	0.95	0.06	0.04	15.93	1.77	0.98	0.05	0.03
Turkey (graduates)	39.89^***^	4.43	0.77	0.10	0.08	42.88^***^	4.76	0.85	0.11	0.06
UK (mostly England)	18.66^*^	2.07	0.95	0.06	0.05	14.45	1.61	0.99	0.05	0.03
UK (Northern Ireland)	6.74	0.75	1.00	0.00	0.03	15.07	1.67	0.97	0.06	0.04

*CFI, Comparative fit index; RMSEA, root mean square error of approximation; SRMR, standardized root mean square residual*.

* *p < 0.05*.

** *p < 0.01*.

*** *p < 0.001*.

As shown in Table 4, the fit indices were acceptable or good in 14 of the 25 samples for benevolent humor. In eight further samples, all fit indices indicated an acceptable fit, with the exception of the CFI. Due to the comparably large number of variables per factor (six), lower CFI values might be found even if the model is correctly specified (see Kenny and McCoach, 2003). Only in three samples (Chile, Taiwan, and the Turkey graduate sample), at least two fit indices were unacceptable. For corrective humor, 20 of the 25 samples showed acceptable or good fit indices, and two showed lower values only in the CFI (China and India). For Latvia, Lebanon, and the Turkey graduate sample, at least two fit indices were unacceptable for corrective humor. Overall, the unidimensionality of benevolent and corrective humor was supported for most samples.

### Measurement invariance across samples, age groups, and gender

Before comparing the factors, correlations, and mean scores, the measurement invariance of the BenCor was tested across samples, age, and gender. Table 5 shows the fit indices of the baseline model (in which the item loadings were allowed to vary freely) with the metric invariance model (in which the item loadings were constrained to be equal across groups) and the scalar invariance model (in which the item loadings and intercepts were constrained to be equal across groups) as well as the changes in the CFI and the RMSEA.

**Table 5:** Fit indices of models assessing metric (fixed loadings) invariance of benevolent and corrective humor across samples.

**Measurement invariance models**	***df***	**AIC**	**CFI**	**RMSEA**	**CFI change**	**RMSEA change**
**BENEVOLENT HUMOR**						
**25 samples (*N* = 7,226)**						
Baseline model	225	144,126	0.95	0.06	–	–
Metric invariance	345	144,103	0.94	0.05	0.014	0.005
**Age (across all samples, 6 age groups^a^)**						
Baseline model	54	147,378	0.95	0.05	–	–
Metric invariance	79	147,352	0.95	0.05	0.001	0.009
Scalar invariance	104	147,562	0.90	0.06	0.053	0.012
**Gender (across all samples^b^)**						
Baseline model	18	147,890	0.96	0.05	–	–
Metric invariance	23	147,891	0.95	0.05	0.003	0.005
Scalar invariance	28	147,964	0.94	0.05	0.003	0.000
**CORRECTIVE HUMOR**						
**25 samples (*N* = 7,226)**						
Baseline model	225	156,578	0.97	0.07	–	–
Metric invariance	345	156,676	0.94	0.07	0.025	0.004
**Age (across all samples, 6 age groups^a^)**						
Baseline model	54	159,516	0.97	0.06	–	–
Metric invariance	79	159,497	0.97	0.05	0.003	0.007
Scalar invariance	104	159,658	0.95	0.06	0.023	0.008
**Gender (across all samples^b^)**						
Baseline model	18	159,731	0.98	0.05	–	–
Metric invariance	23	159,726	0.97	0.05	0.001	0.005
Scalar invariance	28	159,736	0.97	0.04	0.003	0.002

*AIC, Akaike's information criterion; CFI, comparative fit index; RMSEA, root mean square error of approximation*.

a *18–20 years (n = 1,624), 21–24 years (n = 1,981), 25–29 years (n = 1,081), 30–39 years (n = 1,225), 40–49 years (n = 704), 50+ years (n = 580)*.

b *n = 2,906 males and n = 4,312 females*.

As shown in Table 5, the RMSEA changes were < |0.015| for benevolent and corrective humor in each group (i.e., the samples, age groups, and gender). The CFI changes were < |0.01| for the age groups (metric invariance) and gender (scalar invariance), but not for the samples (metric invariance) and the age groups (scalar invariance). Thus, follow-up analyses were conducted for assessing partial measurement invariance, comparing the metric invariance of each of the 12 marker items for the samples and the scalar invariance for the age groups. For the samples, metric invariance was supported for each item, as the CFI change between the baseline model and the metric invariance model was <|0.01| (range |0.001|–|0.008|). For the age groups, the CFI change was also <|0.01| for all items (range |0.000|–|0.008|) with the exception of Item 9 (|0.029|). Thus, partial metric invariance was supported across the samples, partial scalar invariance was supported across the age groups, and scalar invariance was supported for gender. This indicates (a) that benevolent and corrective humor were measured the same way across the different samples, (b) that the factors of the different samples were comparable, and (c) that the mean differences between the age groups and gender could be attributed to mean differences in benevolent and corrective humor. This allows to meaningfully compare the mean-level differences between the BenCor scores across the age groups and gender.

### Factorial validity

The factorial validity of the 12 marker items of benevolent and corrective humor was first tested in an exploratory fashion with Tucker's phi as an index of factor congruence. The 12 marker items were subjected to a PCA with oblimin rotation, in which two factors were extracted. The benevolent and corrective humor factors were then compared with the Swiss student sample, for which the BenCor was originally developed. As shown in Table 3, Tucker's phi indicated factor equality for 14 samples and a fair factor similarity for 8 samples. Lower values were obtained for India and the Turkey graduate sample, for which the extracted BenCor factor was not similar to the comparison sample. The median Tucker's phi value across the 25 samples was 0.95, indicating that the benevolent humor factor showed cross-cultural equality. For the corrective humor factor, 14 samples showed factor equality, and 10 samples indicated a fair factor similarity. With a median of 0.95, cross-cultural factor equality could also be supported for the corrective humor factor.

Next, the factor structure was investigated in CFAs. Both one-factor and two-factor models were estimated based on the 12 marker items, and their fit indices are shown in Table 6.

**Table 6:** Overview of the fit indices of confirmatory factor analyses of the 12 marker items (one-factor and two-factor models) across the 25 bencor samples in the 22 countries.

**Countries**	**One-factor model (*df* = 54)**					**Two-factor model (*df* = 53)**							
	**χ^2^**	**χ^2^/*df***	**CFI**	**RMSEA**	**SRMR**	**χ^2^**	**χ^2^/*df***	**CFI**	**RMSEA**	**SRMR**	***r***	**AVE_Ben_**	**AVE_Cor_**
Austria	332.77	6.16	0.68	0.12	0.10	136.19	2.57	0.91	0.07	0.06	0.47	0.61	0.67
Chile	264.63	4.90	0.67	0.13	0.12	116.83	2.20	0.90	0.07	0.07	0.45	0.62	0.67
China	228.77	4.24	0.66	0.12	0.11	154.78	2.92	0.80	0.09	0.09	0.40	0.57	0.56
Costa Rica	171.18	3.17	0.82	0.11	0.08	135.89	2.56	0.87	0.10	0.07	0.74	0.61	0.70
Croatia	174.31	3.23	0.72	0.08	0.08	104.61	1.97	0.88	0.05	0.06	0.46	0.49	0.53
Germany	204.73	3.79	0.82	0.10	0.09	103.25	1.95	0.94	0.06	0.06	0.59	0.57	0.70
India	98.80	1.83	0.83	0.07	0.07	93.09	1.76	0.85	0.06	0.07	0.81	0.42	0.53
Italian	184.92	3.42	0.71	0.11	0.10	125.67	2.37	0.84	0.08	0.08	0.40	0.52	0.64
Latvia	414.41	7.67	0.70	0.13	0.10	294.12	5.55	0.80	0.11	0.09	0.59	0.62	0.62
Lebanon	185.09	3.43	0.63	0.10	0.08	138.20	2.61	0.76	0.08	0.07	0.43	0.45	0.50
Malaysia	126.15	2.34	0.81	0.08	0.07	92.95	1.75	0.89	0.06	0.06	0.64	0.48	0.57
Malaysia (Terengganu)	118.27	2.19	0.77	0.08	0.08	115.55	2.18	0.77	0.08	0.08	0.80	0.46	0.53
Mexico	117.61	2.18	0.76	0.08	0.08	76.70	1.45	0.91	0.05	0.06	0.53	0.48	0.55
New Zealand	165.11	3.06	0.70	0.10	0.09	108.16	2.04	0.85	0.07	0.06	0.41	0.47	0.63
Poland	348.68	6.46	0.72	0.11	0.09	187.80	3.54	0.87	0.08	0.07	0.50	0.56	0.60
Russia	183.33	3.40	0.61	0.11	0.11	110.20	2.08	0.83	0.07	0.08	0.25	0.48	0.58
Slovakia	222.10	4.11	0.79	0.09	0.07	145.07	2.74	0.88	0.07	0.06	0.63	0.52	0.62
Spain	167.84	3.11	0.74	0.10	0.10	89.27	1.68	0.92	0.06	0.06	0.38	0.51	0.63
Switzerland (general)	286.50	5.31	0.82	0.09	0.07	146.47	2.76	0.93	0.06	0.05	0.65	0.57	0.66
Switzerland (students)	265.07	4.91	0.73	0.11	0.09	78.40	1.48	0.97	0.04	0.04	0.53	0.64	0.67
Taiwan	305.15	5.65	0.75	0.10	0.09	161.41	3.05	0.89	0.07	0.07	0.47	0.56	0.64
Turkey (non-graduates)	197.14	3.65	0.79	0.09	0.07	160.25	3.02	0.85	0.08	0.07	0.70	0.44	0.51
Turkey (graduates)	225.70	4.18	0.65	0.10	0.08	208.00	3.92	0.69	0.10	0.09	0.65	0.53	0.55
UK (England)	201.82	3.74	0.77	0.10	0.08	126.66	2.39	0.89	0.07	0.07	0.59	0.53	0.62
UK (Northern Ireland)	126.42	2.34	0.76	0.08	0.08	84.19	1.59	0.90	0.05	0.06	0.54	0.49	0.58

*CFI, Comparative fit index; RMSEA, root mean square error of approximation; SRMR, standardized root mean square residual; r, correlation between the latent benevolent and corrective humor factors; AVE, square root of average variance explained*.

*All χ^2^ values were significant at p < 0.05*.

As expected, the one-factor model indicated an unacceptable fit in all samples except for India, for which only the CFI was unacceptable. By contrast, the two-factor model showed an acceptable or good fit in all indices (except for the CFI) in 20 of the 25 samples. An unacceptable fit in at least two indices was obtained for China, Costa Rica, Latvia, and the two Turkish samples. These findings mostly support the two-factor structure of the BenCor.

Next, the intercorrelations of benevolent and corrective humor are of interest. Table 3 shows the observed intercorrelations and the factor correlations (from the PCA with oblimin rotation), and Table 6 shows the latent correlations in the two-factor CFA model. In line with the conceptualization of the BenCor, all correlations between benevolent and corrective humor were significant and positive (medium to large effects). The numerically lowest correlations were obtained in Russia, and the highest correlations were obtained in Costa Rica, India, and Malaysia (Terengganu sample). Median correlations were 0.40 for the observed scores, 0.28 for the PCA factors, and 0.53 for the CFA factors. Thus, both the individual samples and the median correlations suggested that benevolent and corrective humor overlap. Still, they can be distinguished from one another, with a median of 28.1% shared true-score variance. Overall, the factorial validity of the BenCor can be supported, albeit to a lesser extent for the samples from India and Turkey (mainly the graduate sample).

Factor analyses (PCA with oblimin rotation and CFA) were also conducted across the full sample of 7,226 participants. The first four eigenvalues in the PCA were 3.67, 1.52, 1.00, and 0.86. Both the scree test and Horn's parallel analysis indicated the retention of two factors, which together explained 43.3% of the variance in the 12 marker items. The loadings and factor intercorrelations are presented in Table 7.

**Table 7:** Loadings and factor intercorrelations of a joint Principal Component Analysis (PCA with oblimin rotation) and a Confirmatory Factor Analysis (CFA with the MLM-Estimator) across the 25 samples.

	**Descriptives**		**PCA**		**CFA**	
	***M***	***SD***	**Ben factor**	**Cor factor**	**Ben factor**	**Cor factor**
**BENEVOLENT HUMOR ITEMS**						
Item 1	5.01	1.39	0.59	−0.04	0.43^***^	–
Item 3	5.10	1.56	0.31	0.30	0.44^***^	–
Item 5	5.50	1.34	0.69	0.06	0.65^***^	–
Item 7	5.31	1.32	0.75	−0.18	0.48^***^	–
Item 9	5.34	1.37	0.59	0.09	0.54^***^	–
Item 11	4.58	1.56	0.59	0.11	0.56^***^	–
**CORRECTIVE HUMOR ITEMS**						
Item 2	4.53	1.72	−0.09	0.67	–	0.51^***^
Item 4	3.97	1.76	−0.03	0.71	–	0.60^***^
Item 6	4.18	1.75	−0.07	0.73	–	0.59^***^
Item 8	4.19	1.63	0.23	0.50	–	0.56^***^
Item 10	3.96	1.73	−0.03	0.77	–	0.68^***^
Item 12	4.18	1.58	0.21	0.56	–	0.61^***^
Factor correlation				0.35		0.58^***^

*N = 7,226*.

*** *p < 0.001*.

As shown in Table 7, each item had its highest loading on the expected factor in the PCA. Main loadings ranged from 0.31 to 0.75 for the benevolent humor factor and from 0.50 to 0.77 for the corrective humor factor. A few cross-loadings were substantial. Item 3 loaded on the corrective factor almost as strongly as on the benevolent factor. By contrast, item 7 had a small negative loading on the corrective humor factor. Items 8 and 12 showed small positive loadings on the benevolent humor factor. In the CFA, all loadings were positive and significant (*p* < 0.001). They ranged from 0.43 to 0.65 for the benevolent humor factor, and from 0.51 to 0.68 for the corrective humor factor. The fit of the two-factor CFA model was unacceptable, with χ^2^ = 1,560.07, *df* = 53, χ^2^/*df* = 29.44, CFI = 0.89, RMSEA = 0.06, and SRMR = 0.05. Still, the two-factor model clearly fitted the data better than the one-factor model (χ^2^ = 3,123.43, *df* = 54, χ^2^/*df* = 57.84, CFI = 0.78, RMSEA = 0.09, and SRMR = 0.07). According to the modification indices, the model fit of the two-factor model could be improved by freeing the loading of item 3 on corrective humor, and the loadings of items 8 and 12 on benevolent humor. The factor correlations were 0.35 for the PCA and 0.58 for the CFA, again indicating a strong overlap, yet no redundancy between the two factors. Thus, although not perfectly aligning with a simple structure, the two factors of benevolent and corrective humor could be clearly separated.

### Discriminant validity

Table 6 also shows the square root of the AVE of the benevolent and corrective humor factors for each sample. Comparing the CFA factor correlations with the square root of the AVE, the Fornell-Larcker criterion was met for benevolent humor in 13 of the 25 samples, and for corrective humor in 18 of 25 samples. The strongest deviations were found for the Indian, the Malaysian (Terengganu), and the two Turkish samples due to their large factor correlations (*r*s ≥ 0.65). Conducting the same analyses across the 25 samples, the square root of the AVE of the benevolent humor factor (0.50) was smaller than the factor correlation (0.58), while the square root of the AVE of the corrective humor factor (0.59) was larger than the factor correlation. Thus, discriminant validity for the benevolent humor factor was only partially supported in terms of the Fornell-Larcker criterion, while the discriminant validity of the corrective humor factor received stronger support.

### Item comparisons across samples

Tables 8, 9 present the means and CITCs of the benevolent and corrective humor items in the 25 samples.

**Table 8:** Minima and maxima of the item means and of the Corrective Item-Total Correlations (CITC) of the benevolent humor items in the 25 samples in the 22 countries.

**Countries**	**Item means**				**CITC**			
	**Min**	**Item**	**Max**	**Item**	**Min**	**Item**	**Max**	**Item**
Austria	4.61	11	5.74	5	0.40	1	0.62	7
Chile	4.61	1	5.72	9	0.39	1	0.63	5
China	4.85	11	5.38	5	0.41	1	0.62	5
Costa Rica	4.76	1	5.24	9	0.36	1	0.59	5
Croatia	4.69	11	5.96	5	0.35	1+9	0.45	5
Germany	4.22	11	5.40	9	0.34	3	0.54	5+7+9
India	4.38	11	5.70	5	0.10	3	0.38	1
Italian	4.41	11	5.83	5	0.27	11	0.48	1
Latvia	4.61	11	5.91	5	0.42	3	0.61	5
Lebanon	4.18	11	5.23	7	0.24	3	0.40	5
Malaysia	4.70	3	5.72	7	0.32	3+7	0.47	5
Malaysia (Terengganu)	4.37	3	6.13	7	0.18	3	0.42	1
Mexico	4.83	11	5.54	5	0.27	1	0.47	9
New Zealand	4.95	11	5.67	5	0.25	1	0.42	7
Poland	4.60	11	5.79	5	0.40	3	0.53	5
Russia	4.59	9	5.51	5	0.21	3	0.50	7
Slovakia	4.58	1	5.61	5	0.32	11	0.55	5
Spain	4.69	11	6.00	9	0.25	3	0.50	5
Switzerland (community)	4.28	11	5.24	9	0.40	3	0.56	9
Switzerland (students)	4.42	11	5.43	3	0.46	1+3	0.65	5
Taiwan	4.79	11	5.35	5	0.35	3	0.56	5
Turkey (non-graduates)	3.85	3	5.66	9	0.28	3	0.54	5
Turkey (graduates)	3.69	3	5.86	9	0.11	3	0.38	5
UK (mostly England)	4.71	11	5.46	5	0.32	1	0.50	5+9
UK (Northern Ireland)	4.96	11	5.81	5	0.27	11	0.54	5

**Table 9:** Minima and maxima of the item means and of the Corrective Item-Total Correlations (CITC) of the corrective humor items in the 25 samples in the 22 countries.

**Countries**	**Item means**				**CITC**			
	**Min**	**Item**	**Max**	**Item**	**Min**	**Item**	**Max**	**Item**
Austria	3.80	4	4.90	2	0.52	8	0.66	4
Chile	4.31	12	5.00	6	0.44	8	0.63	2
China	3.52	8	4.91	2	0.39	2	0.49	4+8
Costa Rica	4.16	2	4.87	8	0.58	2	0.70	12
Croatia	4.07	6	5.17	2	0.34	8	0.55	10
Germany	3.61	4	4.81	2	0.55	2	0.72	10
India	3.91	6	5.47	12	0.35	12	0.51	4
Italian	3.75	8	5.15	6	0.50	4+6	0.64	10
Latvia	3.99	6	4.71	12	0.35	12	0.62	10
Lebanon	2.84	6	4.46	4	0.31	2	0.51	10
Malaysia	3.66	4	4.62	2	0.30	8	0.56	12
Malaysia (Terengganu)	3.09	10	5.19	8	0.33	8	0.49	4
Mexico	3.23	10	4.22	4	0.33	8	0.59	10
New Zealand	4.06	4	4.81	2	0.48	2+8	0.66	10
Poland	4.05	4	4.73	8	0.39	2	0.60	10
Russia	3.30	10	3.90	12	0.13	12	0.66	8
Slovakia	3.89	2	4.34	8	0.41	2	0.64	10
Spain	3.93	2	4.53	4	0.42	8	0.65	10
Switzerland (community)	3.73	4	4.96	2	0.43	2	0.65	4+10
Switzerland (students)	3.78	4	4.86	2	0.52	8	0.64	4
Taiwan	3.75	8	4.74	2	0.37	2	0.62	10
Turkey (non-graduates)	3.17	4	4.37	6+8	0.33	8	0.54	6
Turkey (graduates)	2.78	4	4.55	8	0.32	8	0.50	6+10
UK (mostly England)	3.90	4	4.81	2	0.38	2	0.64	10
UK (Northern Ireland)	4.04	10	5.06	2	0.36	2	0.54	8

As shown in Tables 8, 9, the samples exhibited systematic patterns in terms of the item means and CITCs. First, the means of the benevolent humor items were rather similar across the samples, ranging from 3.69 to 4.96 for the minima and 5.23 to 6.13 for the maxima, while more variation was found for corrective humor, with the minima ranging from 2.78 to 4.31 and the maxima ranging from 3.90 to 5.47. Second, for benevolent humor, item 11 showed the lowest mean in 17 of the 25 samples, while the highest mean was found for item 5 (14 samples). For corrective humor, item 4 showed the lowest mean in 10 of the 25 samples, and the highest mean was found for item 2 (11 samples).

As also shown in Tables 8, 9, none of the items exhibited negative CITCs, indicating that they were all aligned with the total score. Only four samples had CITCs below 0.20, namely India, Malaysia (Terengganu sample), and the Turkey graduate sample for benevolent humor and Russia for corrective humor. The highest values were 0.65 for benevolent humor and 0.72 for corrective humor, indicating that none of the items were redundant. Thus, the psychometric properties of the single marker items seem mostly sufficient. The lowest CITC was found for the benevolent humor item 3 (14 samples), and the highest CITC was found for item 5 (17 samples). For corrective humor, the lowest CITCs were found for items 2 and 8 (11 samples), and the highest CITCs was found for item 10 (14 samples).

### Profile similarities between the samples

**Figure 1:**
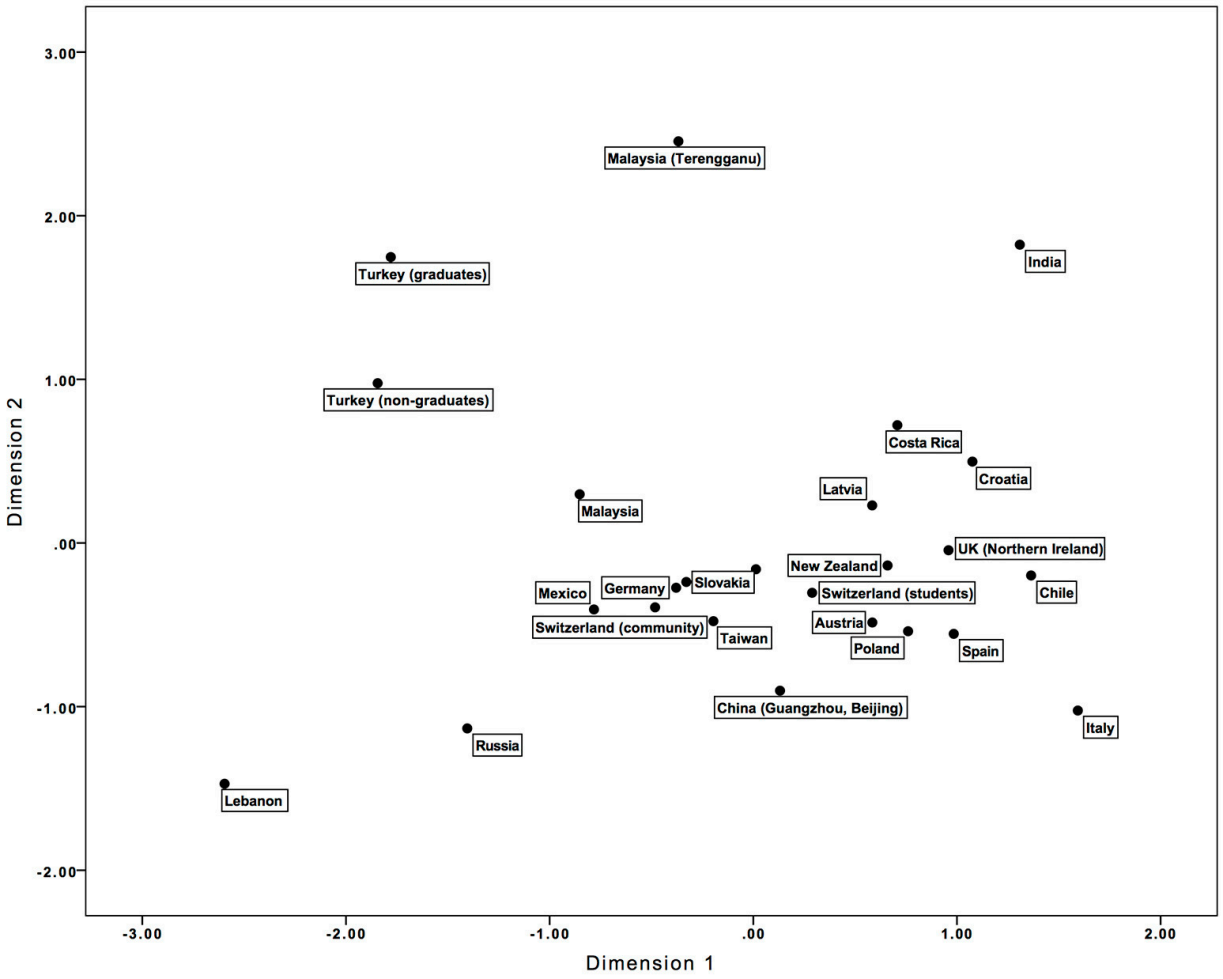
Two-dimensional plot derived from multidimensional scaling of the 12 BenCor items.

The similarities of the samples across the 12 BenCor items were investigated using multidimensional scaling. A two-dimensional solution was chosen (stress function = 0.19, variance explanation 87.4%), which is plotted in Figure 1.

To interpret the solution, the two resulting dimensions were correlated with benevolent and corrective humor and with the single marker items. Dimension 1 correlated strongly with both benevolent [*r*_(25)_ = 0.82, *p* < 0.001] and corrective humor [*r*_(25)_ = 0.91, *p* < 0.001]. That is, Dimension 1 was sensitive to the overall mean differences, contrasting samples with high scores in benevolent and corrective humor (e.g., Italy, India, and Chile) with samples with lower scores (e.g., Lebanon, Russia, and the two Turkish samples). As benevolent and corrective humor showed large positive correlations across the samples, it is not surprising that one dimension of mean-level differences rather than two separate dimensions emerged. Dimension 2 was not significantly correlated with either benevolent or corrective humor (all *p*s ≥ 0.07), and thus correlations at the item level were investigated (for which the significance level was set to 0.01 due to the multiple comparisons). Dimension 2 showed significant correlations with the benevolent humor items 3 [*r*_(25)_ = −0.55, *p* = 0.005] and 7 [*r*_(25)_ = 0.64, *p* = 0.001] and the corrective humor items 8 [*r*_(25)_ = 0.87, *p* < 0.001] and 12 [*r*_(25)_ = 0.67, *p* < 0.001]. Thus, this dimension distinguished samples that were comparably high in three items (7, 8, and 12) and comparably low in item 3. As shown in Figure 1, most samples were rather similar in this dimension, while India, Malaysia (Terengganu region), and the Turkish graduate sample had the highest scores, and Lebanon, Russia, Italy, and China had the lowest scores. This dimension might capture the extent to which item 3 had a corrective connotation and items 8 and 12 had a benevolent connotation, thus potentially decreasing the mean of item 3 and increasing the means of items 8 and 12. In fact, India, Malaysia (Terengganu region), and the Turkish graduate sample showed zero or even negative loadings of item 3 on the benevolent humor factor in the PCA, and items 8 and 12 showed large positive loadings on the benevolent and the corrective humor factor.

**Figure 2:**
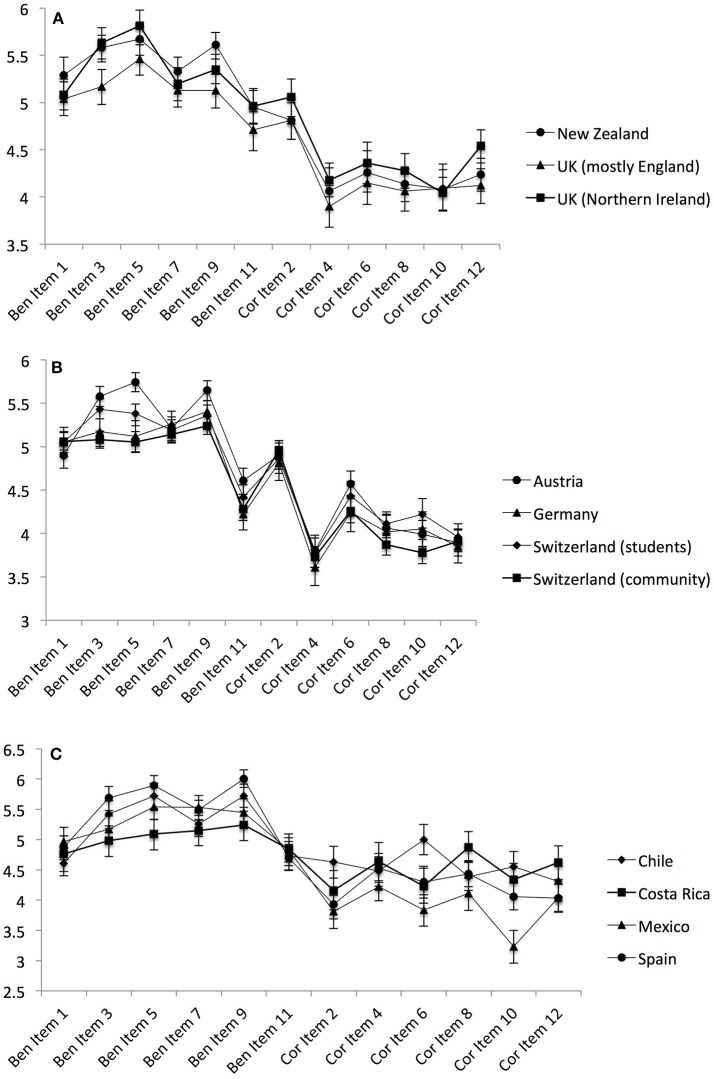
Comparisons of the 12 BenCor items within samples sharing the same language. The upper panel depicts English-speaking samples **(A)**, the middle panel depicts German-speaking samples **(B)**, and the lower panel depicts Spanish-speaking samples **(C)**.

Focusing on the similarity of the countries that shared the same language, item-profile comparisons were conducted. Figure 2 illustrates the item distributions of the English-, German-, and Spanish-speaking samples.

When correlating the samples across the 12 items, a median correlation of 0.97 was found for the English- and the German-speaking countries and a correlation of 0.88 was found for the Spanish-speaking countries. This similarity can also be seen in Figure 2, as the English- and German-speaking countries shared a similar item profile, while the Spanish countries differed more strongly from one another. This similarity was numerically higher than the correlations across the three different languages (0.94 for English and German, 0.80 for English and Spanish, and 0.76 for German and Spanish). Thus, the item mean profiles were most similar for the two Germanic languages, and less similar for Spanish (a Romance language).

Further comparisons were undertaken between the four countries that had two samples each (i.e., Malaysia, Switzerland, Turkey, and the UK). The item-profile correlations within the countries were 0.82 (Malaysia), 0.97 (Switzerland), 0.98 (Turkey), and 0.97 (the UK), indicating a strong similarity within the countries. Importantly, each of these correlations was numerically higher than the correlations between the countries, for which the medians were 0.69, 0.74, 0.66, and 0.77 (for Malaysia, Switzerland, Turkey, and the UK, respectively). This supports the notion that the item profiles of the BenCor were more similar within than between countries.

### Comparisons across age groups and gender

**Figure 3:**
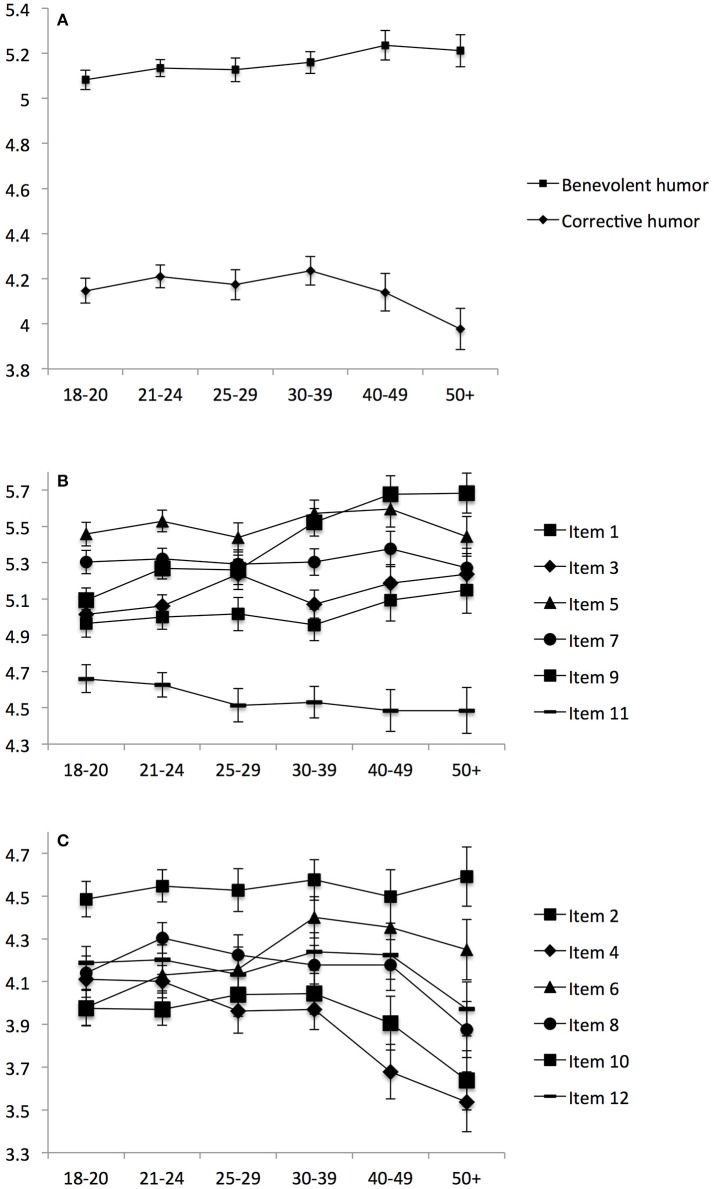
Means with 95% confidence intervals of benevolent and corrective humor **(A)**, the benevolent humor items **(B)**, and the corrective humor items **(C)** for each of six age groups.

Comparisons of the six age groups were conducted with ANCOVAs, controlling for gender. The main effect of age group was significant both for benevolent humor [*F*_(5)_ = 3.98, *p* = 0.001, $\eta_{\text{p}}^{2}$ = 0.002] and corrective humor [*F*_(5)_ = 5.01, *p* < 0.001, $\eta_{\text{p}}^{2}$ = 0.003]. Polynomial contrasts revealed a significant linear trend in benevolent humor (contrast = 0.12, *p* < 0.001), indicating a linear increase with age. For corrective humor, both the linear (contrast = −0.12, *p* = 0.001) and quadratic trends were significant (contrast = −0.15, *p* < 0.001). The means and 95% confidence intervals are shown in Figure 3A.

As shown in Figure 3A, corrective humor tended to increase until the age group of 30–39 years, and then decreased for the age groups of 40–49 and 50+ years. Taking a look at the individual items, ANCOVAs controlling for gender revealed significant main effects for all items (all *p*s < 0.05), except for items 2 (*p* = 0.679) and 7 (*p* = 0.755). Effect sizes were mostly negligible (η_*p*_^2^ < 0.01), with small effects obtained for items 4 (η_*p*_^2^ = 0.011) and 9 (η_*p*_^2^ = 0.023). Significant linear trends were found for the benevolent humor items 1 (contrast = 0.14, *p* = 0.003), 3 (contrast = 0.16, *p* = 0.002), 9 (contrast = 0.53, *p* < 0.001), and 11 (contrast = −0.15, *p* = 0.003). Items 1, 3, and 9 increased with age (in line with benevolent humor), while item 11 tended to decrease with age (see Figure 3B). For corrective humor, linear trends were significant for items 4 (contrast = −0.49, *p* < 0.001), 6 (contrast = 0.27, *p* < 0.001), 8 (contrast = −0.21, *p* < 0.001), 10 (contrast = −0.22, *p* < 0.001), and 12 (contrast = −0.11, *p* = 0.039). Additionally, significant quadratic trends were found for items 4 (contrast = −0.14, *p* = 0.013), 6 (contrast = −0.17, *p* = 0.002), 8 (contrast = −0.22, *p* < 0.001), 10 (contrast = −0.23, *p* < 0.001), and 12 (contrast = −0.12, *p* = 0.015). The negative linear and quadratic trends of Items 4, 8, 10, and 12 were in line with the age trends of corrective humor. Item 6, however, showed a positive linear trend in addition to the negative quadratic trend (see Figure 3C).

Regarding gender differences in benevolent and corrective humor, Table 3 shows the correlations with gender for every sample (with males coded as 1 and females coded as 2). Most correlations with benevolent humor were small and not significant (range −0.14 to 0.11, *Mdn* = −0.04). By contrast, most correlations with corrective humor were negative and significant (range −0.02 to −0.38, *Mdn* = −0.21). When the full sample was analyzed, benevolent humor showed a negligible negative correlation with gender [*r*_(7, 218)_ = −0.05, *p* < 0.001], while corrective humor showed a medium-sized negative correlation [*r*_(7, 218)_ = −0.22, *p* < 0.001]. Thus, gender differences were similar across the samples, and males and females did not substantially differ in their levels of benevolent humor, while males scored higher than females in corrective humor. Comparisons were also conducted for the single items. Significant differences were found for the benevolent humor items 3 and 5, and 11 [*r*s_(7, 218)_ ≤ −0.10, all *p*s < 0.02] and for all corrective humor items [*r*s_(7, 218)_ = −0.11 to −0.18, all *p*s < 0.001], indicating that males always scored higher than females. Thus, the benevolent humor items showed only negligible gender differences, while the corrective humor items consistently showed small gender differences.

## Discussion

The aim of this study was to compare the psychometric properties of the BenCor (Ruch, 2012) across 25 samples from 22 countries. The means and standard deviations differed across the 25 samples, though they all had in common that benevolent humor was more strongly endorsed than corrective humor (around 1 scale point difference). Thus, participants across countries engaged in virtue-related humor, with the benevolent style being more prevalent than the corrective and critical style.

The reliability of both benevolent and corrective humor was supported in most of the samples. Internal consistencies were acceptable, or good, in all samples for corrective humor, while benevolent humor showed somewhat lower values, which were especially low in three samples (India, the Malaysia Terengganu sample, and the Turkish graduate sample). Similarly, unidimensionality was supported in all samples, with the exception of three samples for benevolent (Chile, Taiwan, and the Turkish graduate sample) and corrective humor (Latvia, Lebanon, and the Turkish graduate sample). Thus, the reliability of the sets of marker items of benevolent and corrective humor was either fully or partially supported (except for the Turkish graduate sample). This indicates that the six marker items indeed tapped into a common underlying dimension and that their intercorrelations were positive and sufficient. Thus, despite the brevity of the questionnaire and the rather different contents covered by the marker items (see Ruch and Heintz, 2016), the BenCor seems to be able to measure benevolent and corrective humor reliably across different cultures and languages.

Next, measurement invariance was tested across samples, age groups, and gender. While metric invariance was only partially supported for benevolent and corrective humor across the 25 samples, each of the 12 marker items exhibited metric invariance, thereby allowing comparisons of the factors across the samples (Chen, 2008). For the age groups, metric invariance was supported for benevolent and corrective humor and scalar invariance was supported at the item level (with the exception of item 9). For gender, metric and scalar invariance was fully supported. Thus, both the factors and the means of these groups can be validly compared and are not biased (Chen, 2008). These findings pave the way for comparisons of benevolent and corrective in different countries, in different age groups (e.g., for investigating developmental changes), and for investigating gender differences.

The discriminant validity of the BenCor was partially confirmed using the Fornell-Larcker criterion (Fornell and Larcker, 1981). Specifically, the square root of the AVE of the latent benevolent and corrective humor factors were higher than the correlation between the two factors in 13 and 18 of the 25 samples, respectively. In other words, in more than half of the samples, the variance explanation of the latent benevolent and corrective humor factors in the 12 marker items was higher than the shared variance between the latent factors. Thus, the differences between the two styles of virtue-related humor (i.e., benevolent vs. critical treatment of human weaknesses and wrongdoings) were more pronounced than the similarities (i.e., virtuousness and aiming at the good). Still, the marker items of benevolent humor showed a comparably smaller overlap with their factor, which also fits to the finding that internal consistencies of benevolent humor were lower. Maybe the benevolent humor marker items capture more heterogeneous contents, or maybe the construct itself is more complex. The discrimination among benevolent and corrective humor could be improved by adapting some of the 12 marker items that showed cross-loadings in the PCA and high modification indices in the CFA (i.e., items 3, 8, and 12). This would help to reduce the factor correlation in the CFA. Additionally, more items could be written, which are not merely markers of benevolent and corrective humor, but which represent both constructs comprehensively.

### Factorial validity

Factorial validity for the BenCor was supported both in an exploratory and a confirmatory fashion. First, Tucker's phi indicated that the benevolent and corrective humor factors were fairly similar or equivalent to the Swiss comparison sample (except for the Indian and the Turkish graduate sample). As Tucker's phi is sensitive to differences in item loadings (see Lorenzo-Seva and Ten Berge, 2006), this is in line with the finding of metric invariance of the BenCor; in other words, all samples had similar factor loadings, and thus the meaning and conceptualization of the factors were comparable across samples. Second, CFAs within each sample showed that a two-factor structure fitted the data well in most samples, while the one-factor model did not show an acceptable fit. Also, the true-score correlation between benevolent and corrective humor was much lower than 1 (with a maximum of 64.0% shared true-score variance between the factors). Thus, despite their predictable overlap, benevolent and corrective humor constitute separate factors that capture different forms of virtue-related humor.

Regarding the suitability of the items for the two factors, the PCA across the full sample revealed cross-loadings of items 3, 7, 8, and 12. These differences also aligned well with the profile similarities across the 12 BenCor items, which revealed that the sample similarities were due to the overall mean differences in benevolent and corrective humor (Dimension 1) and due to deviations in 4 items (3, 7, 8, and 12; Dimension 2). Several explanations can be offered for these findings, drawing on both cross-cultural and culture-specific explanations.

Item 3 had similar loadings both on benevolent (0.31) and corrective humor (0.30). This could be due to the low CITCs obtained for this item in 14 of the 25 samples, indicating that this item related less strongly to the total score of benevolent humor than the other items did. It is noticeable that this is the only item that refers to the inclusion of oneself and others when making fun of human weaknesses, while the other items entail the idea of “we, as humans, are all in this together” more directly. Conversely, this item more directly incorporates making fun of human weaknesses (“aiming at”), while the other items rather refer to humor appreciation (e.g., being amused or smiling) or only indirectly entail humor production (treating benevolently). This might shift item 3 to corrective humor, as the latter directly incorporates humor production. Furthermore, PCAs within the samples revealed mismatched loadings (i.e., higher loadings on corrective than on benevolent humor) only for India, the Malaysian Terengganu sample, and for the Turkish graduate sample.

The slightly negative loading of item 7 on corrective humor could be due to it being the only benevolent humor item that explicitly includes the underlying accepting attitude. While both benevolent and corrective humor share detecting weaknesses and treating them humorously, benevolent humor treats them in an accepting manner, while in corrective humor they are not accepted, but instead corrected.

Item 8 had small positive loadings on benevolent humor, which might be due to the softener “gently urge,” which bears resemblance to the benevolent and kind-hearted treatment of weaknesses in benevolent humor. Likewise, “to caricature” might imply a more playful and less critical treatment, and it might additionally be confused with drawing caricatures instead of parodying the wrongdoings physically and verbally. This item had higher loadings on benevolent than corrective humor in six samples (Croatia, India, the two Malaysian samples, and the two Turkish samples).

Finally, item 12 also had small positive loadings on benevolent humor. “Poking fun” is rather soft expression for ridiculing others and might thus have a more entertaining than critical connotation. Likewise, “hoping to improve” focuses on one's optimistic outlook, which might be similar to the humorous outlook entailed in benevolent humor. This item had higher loadings on benevolent than corrective humor in four samples (India, Latvia, Russia, and the Turkish graduate sample).

Several culture-specific differences in the understanding of the items and factors could be hypothesized, which might help to explain some of the deviations found in the factor analyses. For example, in Malaysia (Terengganu region), several informal interviews suggested that corrective humor seems to have an inherent benevolence, as close bonds exist between people and informing others about their wrongdoings in a respectful, but also humorous manner is expected and encouraged within friendships. Thus, the virtuous aspect of corrective humor might be stronger in this culture, also distinguishing this sample from the general Malaysian sample. In the Croatian, Indian, and Latvian contexts, corrective humor might not be employed at the societal level very often, perhaps because people do not feel that they can produce a change, and people might thus rather adjust than try to change the conditions with satirical remarks. Also, corrective humor might not only serve to correct transgressions, but it might also serve as a coping mechanism by venting one's feelings in making public humorous remarks about things that go wrong, independent of whether an improvement can actually be achieved or not. For the Russian context, existential freedom and implicit creative potential might be valued. Thus, there would be less need to correct rule breaking, as it would be considered a manifestation of free will, which might even arouse some sympathy. These hypotheses on cultural differences in benevolent and corrective humor should be systematically explored in future studies.

### Age and gender differences

Going beyond cross-cultural comparisons, age and gender differences were explored. Although the differences found in these demographic variables were negligible or small, they still fitted well to the conceptualization of benevolent and corrective humor. Benevolent humor, especially item 9, showed linear increases with age. Item 9 (“Humor is suitable for arousing understanding and sympathy for imperfections and the human condition”) might have had the strongest age effects for two reasons. First, it entails an attitude rather than showing humor directly. This is in line with findings that agreeableness increased with age, and extraversion and openness decreased with age (see Marsh et al., 2013). Specifically, the benevolent, serene, and accepting attitude underlying benevolent humor might increase, while making humorous remarks and enjoying humor in general might rather decrease in line with decreases in extraversion and openness (see Craik et al., 1996; Köhler and Ruch, 1996; Martin et al., 2003; Nusbaum et al., 2017). A second explanation takes into account the lack of scalar measurement invariance found for this item across age groups. Having different intercepts in the different age groups might lead to over- or underestimations of the means of specific groups, thus potentially reflecting bias instead of true mean differences (see Chen, 2008). For example, if older age groups had higher intercepts and younger age groups had lower intercepts than middle-aged adults, the means of the older groups might be overestimated and those of the younger groups underestimated.

For corrective humor, decreasing linear and quadratic trends were found. Thus, middle-aged adults engaged most often in this type of humor, followed by younger adults, with the lowest scores obtained for older adults. This developmental trajectory also fits to the increase in agreeableness and the decrease in extraversion and openness with age (Marsh et al., 2013), which would potentially explain the negative linear trend observed. The curvilinear trend was similar to the negative quadratic relationship of conscientiousness with age. Potentially, people who are more conscientious care more about what is right and wrong (i.e., they might have a stronger moral compass), which could potentially increase their levels of corrective humor. An alternative explanation could be that middle-aged adults are faced with situations in which they can employ corrective humor more often (e.g., at the workplace), and they might also believe that their humorous remarks can improve the conditions.

Regarding gender differences, men consistently scored higher in corrective humor than females, while only negligible gender differences were found for benevolent humor. This is consistent with other studies that found gender differences mostly for critical or affective forms of humor (such as sexual and aggressive humor; Martin et al., 2003; Lampert and Ervin-Tripp, 2007). By contrast, gender differences in the sense of humor and in humor as character strength (which was more strongly aligned to benevolent than to corrective humor; Ruch and Heintz, 2016) were usually small or negligible (Lampert and Ervin-Tripp, 2007; Heintz et al., 2017).

### Limitations and directions for future studies

The present study serves as a starting point for more extensive cross-cultural research and applications in the area of humor and particularly virtue-related forms of humor. However, several limitations can be noted. First, although the 25 samples allowed some cross-cultural comparisons, analyses at the sample level were limited due to the low statistical power. Thus, substantially increasing the number of samples is needed for additional comparisons, like correlating the samples' BenCor scores with other sample-specific indicators, such as culture dimensions (Hofstede, 2001), sample gelotophobia and character strengths scores (Proyer et al., 2009; McGrath, 2015), and broad personality traits (Schmitt et al., 2007). Additionally, employing more samples would allow more detailed comparisons of samples from the same region vs. different regions (e.g., cities vs. rural environments, tribes of indigenous people) in the same country, from neighboring vs. adjacent countries, and from different language versions within the same country and across countries. This would help to disentangle the role of the local and national cultural norms and the influence of different languages (see Park et al., 2006; Proyer et al., 2009; McGrath, 2015) in determining similarities in the BenCor. For example, it was suggested that more collectivistic cultures, in comparison to more individualistic cultures, place higher importance on maintaining others' faces and thus rather avoid than dominate conflicts (Ting-Toomey et al., 1991). Thus, openly voicing criticism (whether humorously or not) might be less acceptable in collectivistic cultures such as China, Taiwan, and Japan, which would suggest that (a) the mean values of corrective humor would be lower, (b) corrective humor might be less seen as related to virtue, and consequently (c) the correlation between benevolent and corrective humor might be lower than in more individualistic cultures such as the United States. These hypotheses could be tested in future studies that systematically compare countries that differ in their collectivism and individualism scores.

Second, although the 12 marker items worked well in a majority of the samples, one could still think of slight adaptations that might shift them more strongly to the factor they belong to and that decrease the overlap between the two factors. For item 3, two changes are proposed, replacing “is aimed at” with “deals with” to make it less critical, and replacing “I include both myself and others” by “I refer to humans in general, including myself” (suggested rephrased item 3: “When my humor deals with human weaknesses, I refer to humans in general, including myself”). Item 8 could be simplified by replacing “caricature in a funny way” (which might be hard to understand or might be potentially misunderstood) by “making fun of”, and by removing the term “gently” (suggested rephrased item 8: “I make fun of my fellow humans' wrongdoings to urge them to change”). Finally, item 12 could be made more corrective by replacing “poking fun” with “ridiculing” and by removing “hoping” (“If the circumstances are not as they actually should be, I ridicule these moral transgressions or societal wrongdoings to improve them in the long term”). The psychometric properties of these adapted marker items will be tested in future studies. If they are found to be superior to the existing marker items, these might be replaced in order to optimize the BenCor.

Third, the present study focused mainly on the psychometric properties of the BenCor and the need for separating the two concepts. Future studies can investigate their differential criterion validity in different countries. Thus far, only German-speaking countries have been investigated (Ruch and Heintz, 2016; Ruch et al., 2018a,b). For example, the BenCor could be related to different positive psychological variables such as subjective well-being (Diener et al., 2009), positive emotions (Shiota et al., 2017), and resilience (Masten et al., 2009) to establish the nomological network of benevolent and corrective humor. Replicating this nomological network in different countries would be an important task for future cross-cultural research on virtue-related humor. These studies could also include already established predictors of these outcomes (such as broad personality traits) as well as measures of the sense of humor and mockery to determine the incremental validity and unique contribution of the BenCor to the positive-psychological outcomes. Furthermore, gelotophobia (the fear of being laughed at) should be assessed as a control variable, as individuals with high scores have been shown to react less positively and more negatively to enjoyable emotions that elicit laughter (Platt et al., 2013; Ruch et al., 2015) and to have problems with intrapersonal emotion-related skills more generally (Papousek et al., 2009).

Fourth, in terms of age, the developmental trajectories of both benevolent and corrective humor deserve future studies to understand the underlying reasons for the age differences. Also, longitudinal investigations (for an overview, see Collins, 2006) would be needed to be able to distinguish among true developmental changes and cohort differences.

## Conclusions

Overall, the present study supported the usefulness of the BenCor, a set of 12 marker items that assesses benevolent and corrective humor, for 22 different countries. This is especially remarkable as these historical concepts are rather complex and sophisticated, yet they could be recovered in different cultures and languages, allowing the accumulation of research findings across different cultures—at least the ones investigated so far. Thus, this study lays the foundations for closing the virtue gap in humor by providing an economic and reliable means of integrating benevolent and corrective humor in research across the world. Once the BenCor is sufficiently validated, it can fruitfully supplement existing humor applications in various areas, for example at the workplace (e.g., Robert, 2016), in clinical settings (e.g., Konradt et al., 2013), and in positive interventions (e.g., Wellenzohn et al., 2016a,b).

## Ethics statement

The studies were carried out in accordance with the recommendations of the local ethical guidelines of the committees of the following institutions: Catholic University in Ružomberok, HELP University, Indian Institute of Technology Delhi, Lebanese University, National Taiwan Normal University, Saint Petersburg State University, Universidad Andrés Bello, University of Granada, University of Latvia, Universiti Malaysia Terengganu, Universidad de Monterrey University of Rijeka University of Waikato, University of Wolverhampton, and University of Zurich. All participants provided either online or written informed consent in accordance with the Declaration of Helsinki.

## Author contributions

WR and SH conceived the study and organized the data collection. SH conducted the data analyses and drafted the manuscript. All authors were involved in the data collection and revisions of the manuscript.

### Conflict of interest statement

The authors declare that the research was conducted in the absence of any commercial or financial relationships that could be construed as a potential conflict of interest.
